# Utilisation of Non-Edible Source (*Pongamia pinnata* Seeds Shells) for Producing Methyl Esters as Cleaner Fuel in the Presence of a Novel Heterogeneous Catalyst Synthesized from Waste Eggshells

**DOI:** 10.3390/molecules26195772

**Published:** 2021-09-23

**Authors:** Ala’a H. Al-Muhtaseb

**Affiliations:** Department of Petroleum and Chemical Engineering, College of Engineering, Sultan Qaboos University, Muscat 123, Oman; muhtaseb@squ.edu.om

**Keywords:** heterogeneous catalyst, waste eggshells, caesium oxide, nonedible biomass, biodiesel, process optimisation

## Abstract

Waste eggshells were considered for synthesising a precursor (CaO) for a heterogeneous catalyst, further impregnated by alkali caesium oxide (Cs_2_O). The following techniques were used to characterise the synthesised catalysts: X-ray Diffraction (XRD), Scanning Electron Microscopy (SEM), Energy-dispersive X-ray spectroscopy (EDS), X-ray photoelectron spectroscopy (XPS) and Temperature Programmed Desorption (CO_2_-TPD). The synthesised catalyst revealed its suitability for transesterification to produce biodiesel. The biodiesel production process was optimised, and it showed that the optimal biodiesel yield is 93.59%. The optimal set of process parameters is process temperature 80 °C, process time 90 min, methanol-to-oil molar ratio 8 and catalyst loading 3 wt.%. It has been found that the high basicity of the catalyst tends to give a high biodiesel yield at low methanol-to-oil ratio 8 when the reaction time is also less (90 min). The fuel properties of biodiesel also satisfied the standard limits defined by ASTM and the EN standards. Thus, the synthesised catalyst from waste eggshells is highly active, improved the biodiesel production conditions and PPSS oil is a potential nonedible source.

## 1. Introduction

In this era of competition, developed and underdeveloped countries are expanding their industrial zones to enhance the production of various goods, which ultimately boost the economy [1,2]. If the statistics were triggered, it increased exponentially, causing an increment in the energy demand. Fossil fuels are the primary source for fulfilment, since their exploration is thought to diminish in the coming 50 years [3,4,5]. Moreover, there are other challenges, such as instability in prices, depletion of reserves and environmental issues related to combustion emissions [6]. To tackle all these issues associated with fossil fuel reserves and their combustion, researchers must dig out potential alternative sources for energy production in the industrial sector and meet the increasing demand for the transportation sector [7,8].

The rummage for alternative energy leads to potential sources, including wind energy, solar power and biomass-derived fuels. Among all the alternative sources, biomass-derived fuels are more diversified in energy capacity, consumption, storage and transportation [2,9]. One obsolete method regarding biomass consumption as an energy source was its direct combustion, by which 70% of the energy content is wasted [10]. Over time, several techniques have developed to utilise biomass for energy production through several routes, including gasification, pyrolysis and oil extraction. It has been reported that oil extracted from a biomass can be used as a fuel in the forms of blends with fossil fuel, as well as used directly [11,12,13,14].

A transesterification process can achieve the transformation of biomass-derived oil to biodiesel fuel. It is a reaction between oil (triglycerides) and short-chain alcohol in the presence of a suitable catalyst [15]. The product of this reaction is the formation of alkylated esters, which are biodiesel and glycerol. Short-chain alcohol refers to methanol, ethanol or butanol. Ethanol and butanol showed some issues while using them as reactants with triglycerides, including by-product separation and the purity of product biodiesel [16,17]. Thus, methanol is the most used alcohol worldwide for biodiesel production through transesterification. Biodiesel offers several advantages over fossil diesel; it is a biomass-derived product; therefore, it is thought to be biodegradable. Based on the prototype results, it has been reported that it emits significantly few carcinogenic agents when combusted in conventional diesel engines compared to conventional fossil diesel and does not contribute to emitting greenhouse gases, thus leading to clean and eco-friendly fuel [18,19].

Initially, biodiesel production started by transforming the lipids extracted from edible sources, mostly vegetable oils [20,21]. Thus, over time, edible oil consumption in this matter may cause food sacristy, which ultimately ended to avoid food vs. fuel controversy. Moreover, when edible oil is used for biodiesel production, it leads to high-cost end product biodiesel that reported that 70% of the biodiesel cost is accounted for from its feedstock [20,22]. Thus, to avoid these controversies regarding biodiesel cost, nonedible sources are mainly researched and used in a lab and on the commercial scale for biodiesel production. Nonedible feedstocks are mostly referred to as agricultural waste or barren land agricultural products with less or no costs and are abundantly available. Most commonly reported among many nonedible feedstocks are Jatropha, rubber seeds and date seeds.

Similarly, a ubiquitous tree known as *Pongamia pinnata* is abundantly available in various parts of the world [23]. A shell covers *Pongamia pinnata* seeds, and after drying the seeds, the shell becomes hard enough to be ground into a powder form to be subjected to oil extraction [24]. *Pongamia pinnata* seeds shells (PPSS), which occupy almost 50 wt.% of seeds, can be a potential source of nonedible oil for biodiesel production.

The catalyst is a crucial component for a transesterification reaction to produce biodiesel. A basic nature catalyst, either alkali hydroxides or alkaline oxides, is considered for biodiesel production [1,3,25,26]. Conventionally, alkali hydroxides, which include potassium and sodium hydroxides, were used as a highly efficient catalyst. Despite high-efficiency alkali hydroxides in the homogeneous phase, reactants and products have difficulty separating the catalysts from products. Thus, due to the one-time usage of a homogeneous catalyst, the process tends to become cost-intensive and causes the cost of biodiesel to increase. Heterogeneous catalysts have been studied and explored widely since the twentieth century to overcome the drawbacks mentioned above. Among all heterogeneous catalysts, the most commonly used are alkaline oxides (CaO, MgO and BaO) and transition metal oxides (ZrO_2_, MnO, ZnO and MoO) used either directly or in their modified forms [27]. However, a heterogeneous catalyst tends to overcome the issue that it is reusable and reduces the cost of producing biodiesel. Despite this, the utmost need for commercialisation is to make the biodiesel production process more economical—a catalyst with high basicity, reducing the reaction time and methanol-to-oil molar ratio.

Moreover, materials derived from waste, such as eggshells consisting of calcium carbonate, can be calcined to get calcium oxide CaO, considered a highly basic material [28,29]. However, lime modified further with high basicity can help get a highly efficient catalyst for biodiesel production. Thus, first, group oxides can increase the alkalinity, and caesium oxide can be potential candidates [30,31]. Therefore, lime (CaO) obtained from waste eggshells modified with caesium oxide can be a potential active catalyst with high basicity for biodiesel production [32,33].

The present research work reports the synthesis of a highly efficient heterogeneous catalyst derived from waste eggshell and impregnated further with caesium oxide and used for biodiesel production, which is novel of its kind and not reported before, as per the authors’ knowledge. The characterisation of the synthesised catalyst revealed its high activity towards the transesterification reaction for biodiesel production. The PPSS was used as a source of oil that is transformed into biodiesel in a synthesised novel heterogeneous catalyst. The biodiesel was analysed, revealing that PPSS could be a potential alternative source for biodiesel production.

## 2. Materials and Methodology

### 2.1. Materials

*Pongamia pinnata* seeds shells (PPSS) were collected from the local farm (Muscat, Oman); the shells were removed. The shells were washed, followed by sun-drying for 48 h and then in the oven for 8 h at 120 °C. The PPSS shells were then ground to a fine powder and applied to the extraction process for oil extraction in the Soxhlet apparatus, adopting the standard method Am 2-93. Waste eggshells were collected from the university café, then washed, and the thin inner layer was removed, leaving pure shells, which were then dried in an oven for 12 h at 120 °C. All other chemicals, including n-hexane, methanol and caesium nitrate, were of reagent grade purchased from Sigma-Aldrich (Germany).

### 2.2. Catalyst Synthesis

Dried waste eggshells are considered a source of calcium oxides. The dried shells were ground into a fine powder and placed in a muffle furnace for calcination in an inert atmosphere. The furnace was controlled at 900 °C for 4 h and started at 4 °C/min. The resultant material was allowed to cool down and expected to be pure lime CaO (EC), further impregnated with alkali caesium oxide (Cs_2_O) by wet impregnation. An initially known amount (100 g) of pristine lime was taken in the beaker, and a solution of alkali (Cs) salt was poured into lime powder dropwise with continuous stirring. The mixture of lime and alkali salt was left 12 h. Then, the mixture is placed in the oven to remove the outer surface water content at 120 °C for 12 h; then, the resultant material was calcinated in a muffle furnace in an inert atmosphere at 500 °C for 4 h with a rate of 4 °C/min. Once the time was completed, the resultant material was allowed to cool to room temperature in a desiccator. A different sample of catalyst was prepared by changing the composition of caesium solution (10, 15, 20 and 25 wt.%), and the samples were named, respectively, EC-10, EC-15, EC-20 and EC-25.

### 2.3. Catalyst Characterisation

The synthesised catalysts were characterised by the following techniques (powdered X-ray diffraction (XRD) by PANalytical, Xpert PRO, USA, which has a rotatable anode, and Cu Kα radiation, field emission scanning electron microscopy (FESEM) and energy-dispersive X-ray spectroscopy (EDS) by JEOL JSM-7800F, Japan. The temperature programmed desorption (TPD) by Thermo Finnigan of the initial material was treated with a continuous nitrogen flow while heating it from 50 to 150 °C for 1h to make it moisture-free, followed by the adsorption of carbon dioxide after that sample heated to 950° in the presence of helium as a carrier. X-ray photoelectron spectroscopy (XPS) by Omicron Nanotechnology, Erlangen, Germany was used to determine its nature.

### 2.4. Biodiesel Production and Its Quality Determination

Biodiesel is produced using PPSS oil transesterification in the presence of a synthesised novel catalyst. All experiments were conducted in a three-neck reaction vessel placed on a hotplate while stirring. The temperature was also controlled from the same heating source by setting the thermocouple with the reaction mixture. All the experiments were conducted as per the plan developed using the experiment (DOE) software 12.0 (State-Ease, Minneapolis, MN, USA). The experimental procedure was developed based on the four independent process parameters’ (temperature, time, methanol-to-oil molar ratio and catalyst loading) specific ranges, as shown in Table 1. It was noticed that the experimental plan, as shown in Table 2, consisted of thirty experimental runs; within which, six were repeated runs on the centre points to check the errors. Each experimental run was conducted thrice to assess the errors in the results. Based on the reaction condition, the initial starting reaction catalyst was well-mixed with methanol. The mixture was poured into the heated (temperature based on specific run reaction conditions) oil. Once the reaction was stopped, the mixture was allowed to cool and pour down in a separatory funnel and left for 24 h for a distinct separation of product biodiesel and by-product glycerol. The mixture was separated into two layers, the upper known as biodiesel and the bottom as by-product glycerol. After separating biodiesel from glycerol, it was washed with warm water and stored in an airtight bottle. To have a statistical analysis, ANOVA was used and based on which one can predict how significant the model (shown in Equation (1)) was with the experimental data.

(1) $$Y=\beta_{o}+\overset{k}{\underset{i=1}{\sum }}\beta_{i}X_{i}+\overset{k}{\underset{i=1}{\sum }}\beta_{ij}X_{i}^{2}+\overset{k}{\underset{i>j}{\sum }}\overset{k}{\underset{j}{\sum }}\beta_{ij}X_{i}X_{j}+e$$

*Y* denotes biodiesel yield *β* as a regression coefficient, *X_i_* and *X_j_* denote coded variables (*i* and *k)* denotes how experimental parameters are, and the error is *e*. Moreover, for a quality analysis of the produced biodiesel, several analyses were used, such as for composition gas chromatography-mass spectroscopy GC-MS and for fuel properties, several tests (acid value (D664 ASTM), density (5002 ASTM), cloud point (D2500 ASTM), cetane number (D613 ASTM), cold filter plugging point (D6371 ASTM), kinematic viscosity (D445 ASTM), pour point (D97 ASTM) and flashpoint (D93 ASTM)), were done. The reusability of the catalyst was determined by separating the catalyst after each experimental run and then washing it with methanol thoroughly and drying it at 120 °C for 4 h and using it for the next experiment. This procedure was repeated for ten experimental runs, and based on this, the reusability of the catalyst was reported.

## 3. Results and Discussion

### 3.1. Catalyst Characterisation

#### 3.1.1. X-ray Diffraction Analysis

**Figure 1:**
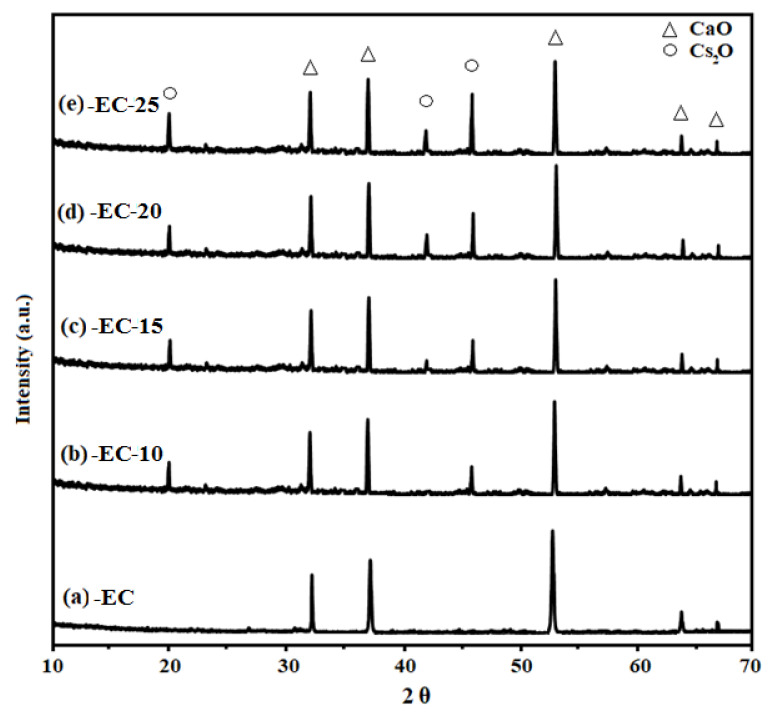
Catalyst analysis by XRD: (**a**) lime (CaO) derived from waste eggshells, (**b**) lime modified by 10 wt.% of caesium oxide, (**c**) lime modified by 15 wt.% of caesium oxide, (**d**) lime modified by 20 wt.% of caesium oxide and (**e**) lime modified by 25 wt.% of caesium oxide.

The lime (CaO) phase derived from the waste eggshell and its modified forms was determined by the X-ray diffraction analysis (XRD) (X’Pert Pr, Panalytical, UK), as shown in Figure 1. The XRD pattern for lime had four definite diffraction peaks (2θ = 32.2°, 37.4°, 53.8° and 67.1°) [29,34], which matched with the library (JCPDS File No. 00-37-1497), presenting pure CaO that was attributed to eggshells, which consist of CaCO_3_ and decompose well to CaO after treating at 900 °C. The sharp peaks present in the diffractogram presenting CaO revealed high material in the crystalline cubic phase. Further on, XRD patterns for lime modified with alkali oxide (Cs_2_O) in different fractions are also given in Figure 1. It can be noticed that the diffractogram presenting the lime modified with 10 wt.% of Cs_2_O showed two additional peaks at 20.1° and 46.1°, which confirmed the presence of Cs_2_O [31]. However, it has been noticed that, when the quantity of alkali oxide was increased in the binary mixture of Cs_2_O–CaO, the strength of the peaks representing the alkali oxide increased; moreover, another small additional peak appeared in the pattern at 42.2°.

The conventional peaks in the diffractogram representing Cs_2_O reported in the literature are consistent with the figure’s data. Based on the peaks present in diffractograms for pure lime and its modified forms, pure oxides (Cs_2_O and CaO) showed that, in the binary mixture of Cs_2_O–CaO, no additional homogeneous phase was formed. Thus, this observation that no new homogeneous phase of Cs_2_O–CaO formed was due to the unique metal ions ionic radii. The increase in the strength of the Cs_2_O peaks when its quantity is increased in a binary system can be related to the fact that, initially, when the Cs_2_O amount is less, some of it goes into the pore of lime, and upon increasing its quantity, it starts appearing on the surface of lime, resulting in a higher peak strength. Therefore, it can be concluded that waste eggshell is treated successfully to target the material (CaO), and further on, its modification is done successfully too, as there are additional peaks present in the parent material diffractogram.

#### 3.1.2. Scanning Electron Microscopy and Energy Dispersive Spectroscopy Analysis

**Figure 2:**
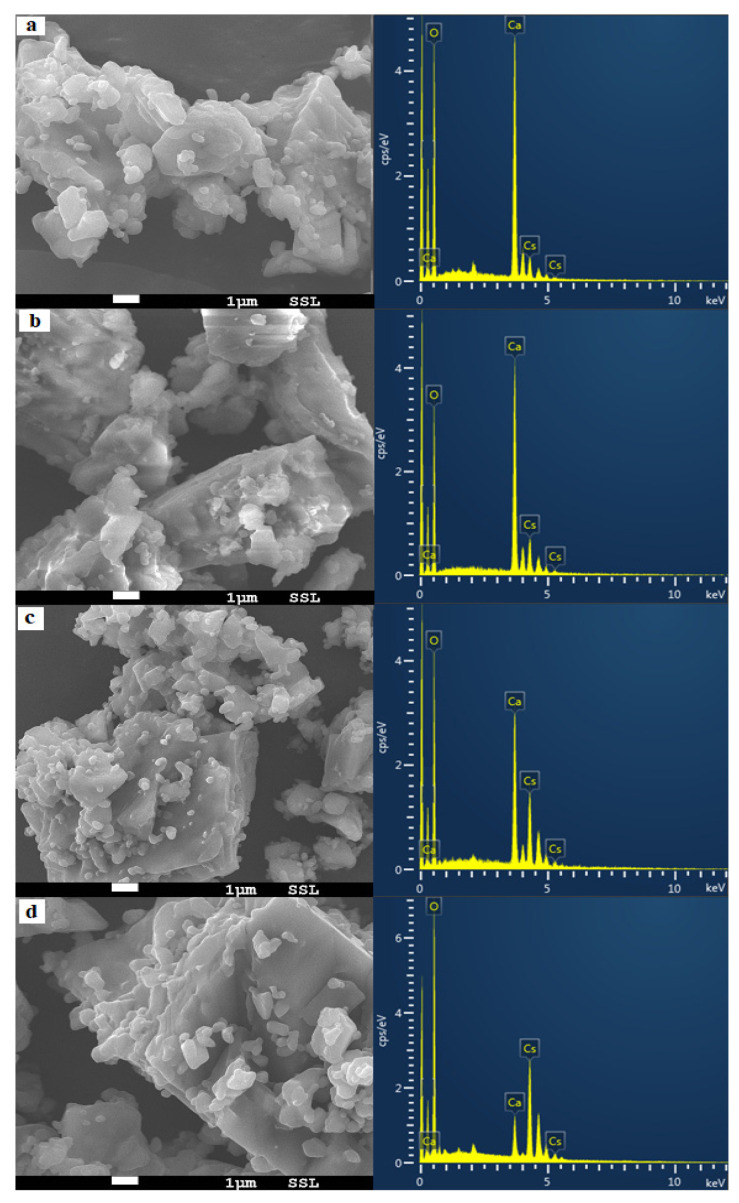
Catalyst analysis by SEM and EDS: (**a**) lime (CaO) modified by 10 wt.% of caesium oxide, (**b**) lime modified by 15 wt.% of caesium oxide, (**c**) lime modified by 20 wt.% of caesium oxide and (**d**) lime modified by 25 wt.% of caesium oxide.

The surface morphology of the synthesised catalysts was analysed based on the results obtained from scanning electron microscopy SEM images, as shown in Figure 2. It can be noticed that the SEM image of all the samples showed that the particles were of irregular shape. Moreover, the EDS results were reported beside each sample’s SEM image, which showed that both oxides were present in each sample, thus tending to the observation that Cs_2_O was successfully impregnated with lime derived from eggshells. SEM images observed that the binary system Cs_2_O–CaO resultant material had a certain number of pores and attachments of particles, and these results are consistent with XRD. Moreover, the SEM images also showed that the catalyst possessed dense bulky particles with active alkali oxide distribution heterogeneously. Thus, based on the surface morphology, it can be considered that catalysts synthesised can offer suitable activity towards the transesterification reaction for biodiesel production.

#### 3.1.3. Surface Characterisation: X-ray Photoelectron Spectroscopy

**Figure 3:**
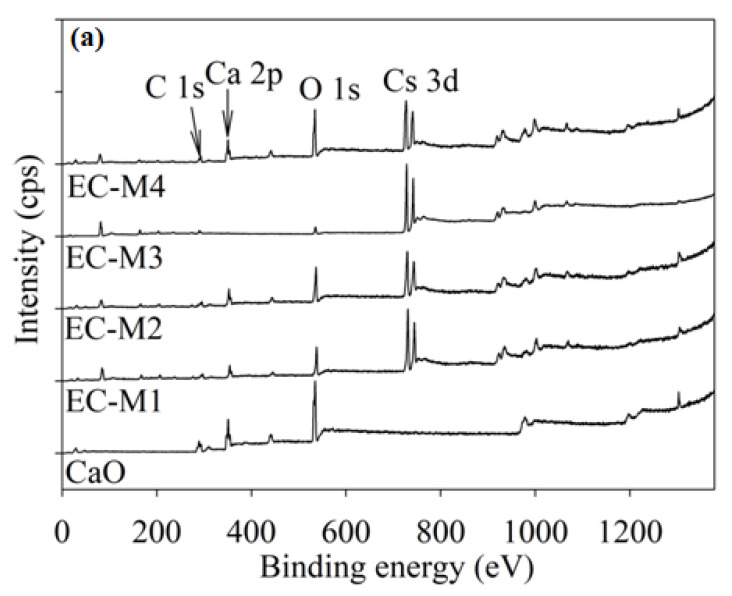

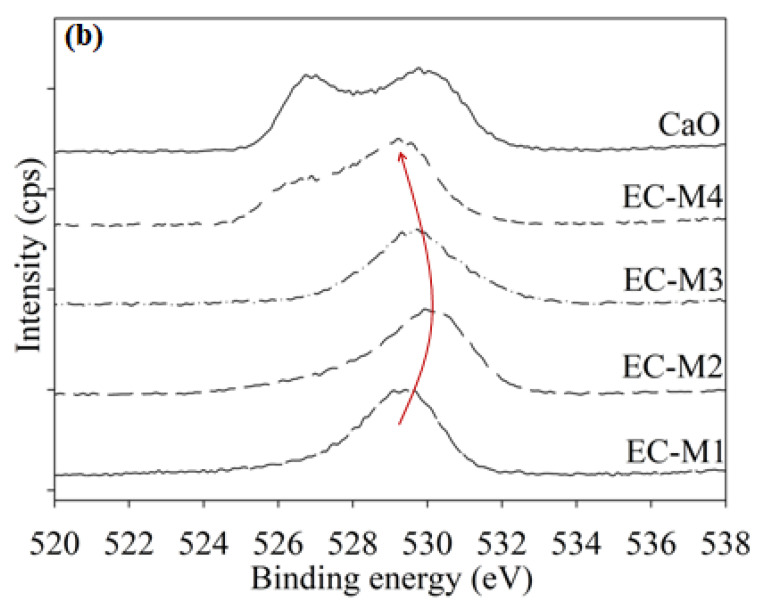
XPS spectra of (**a**) the survey spectra for CaO and CsO_2_-modified catalysts. (**b**) High-resolution O 1s peaks of the CaO and CsO_2_-modified catalysts.

The surface nature of parent material CaO and synthesised catalysts EC-10, EC-15, EC-20 and EC-25 were characterised using the X-ray photoelectron spectroscopy technique. Figure 3a shows the survey spectra of CaO and the EC-10, EC-15, EC-20 and EC- catalysts where the expected elements of Ca, O and C for the CaO catalyst and Cs, Ca, O and C for the synthesised catalysts were detected on the surface. All modified catalysts displayed the presence of caesium, where the calcium content decreased after modifying with caesium. However, the presence of calcium on the EC-20 catalyst surface was the minimum, where caesium was the maximum; thus, the existence of oxygen on the surface was also decreased (due to the oxidation nature of Cs_2_O). This could be due to the nonuniformity distribution of Cs_2_O on the parent CaO surface.

All in all, the presence of caesium on the parent materials exhibited the successful synthesis of Cs_2_O–CaO catalysts. Figure 3b shows the core level O1s peaks for the parent CaO and EC-10, EC-15, EC-20 and EC-25 catalysts. For the parent CaO catalyst, two distinct peaks were observed at 527 eV and 530 eV, respectively. The binding energy of the core level O 1s shifted to a lower value for the EC-10 catalyst compared with the parent CaO (530 eV → 529.4 eV) but shifted back to the higher value (very similar to the parent CaO of ~530 eV) for the EC-15 catalyst. The EC-20 catalyst showed the binding energy shifting back to a lower value (~529.8 eV), while the EC-25 catalyst showed a very similar binding energy value (~529.3 eV) EC-10 catalyst. These binding energies shifting exhibited an interaction between CaO and Cs_2_O to form a Cs_2_O–CaO composite catalyst. Besides, for the EC-25 catalyst, the O1s peak showed an extra shoulder peak at 526 eV, which might come from the parent CaO catalyst (see Figure 3b).

#### 3.1.4. CO_2_-Temperature Programmed Desorption Analysis

The basic strength was quantified using the carbon dioxide temperature-programmed desorption CO_2_-TPD technique, and the results are shown in Table 3. It can be noticed that CaO possesses a certain number of basic sites; however, by its modification with Cs_2_O, it was increased. The increment in the basic strength of the binary system Cs_2_O–CaO was due to the basic nature of Cs_2_O. The synthesised catalyst was highly basic with the variations in the strength, which were evaluated.

### 3.2. Catalyst Evaluation

**Figure 4:**
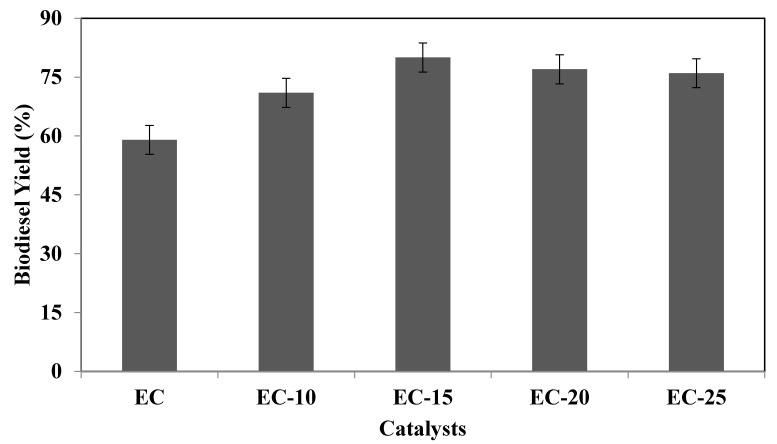
Catalyst evaluation selects the most suitable catalyst for transesterification to produce biodiesel (temperature—70 °C, oil-to-methanol molar ratio—6, time 60 min and catalyst loading—2 wt.%) among all the synthesised catalyst samples.

The synthesised catalyst was in the crystalline phase with irregularly shaped particles and was highly alkaline. A complete characterisation confirmed that the synthesised catalyst was highly suitable to support the transesterification reaction for biodiesel production. To evaluate the most appropriate catalyst among them all, the transesterification reactions of PPSS oil were done to produce biodiesel. Among all the synthesised catalysts, the most promising biodiesel yield was given by the EC-15 catalyst, as shown in Figure 4.

Moreover, in terms of basicity, as shown in Table 3, EC-15 possessed a certain high amount (6.46 ± 0.11) of active basic sites, which ultimately resulted in a high biodiesel yield. Thus, it can be concluded that EC-15, when used as a catalyst for the transesterification of PPSS oil to produce biodiesel, tends to be the most suitable, as it gave the highest biodiesel among all the synthesised catalysts. Therefore, EC-15 will be further used for the parametric study to optimise the processing condition (temperature, time, catalyst loading and methanol-to-oil molar ratio).

### 3.3. Model Prediction and Statistical Analysis

To develop a mathematical model based on the experimental data generated in current research work, RSM was used. Four independent variables were considered: process temperature, time, the molar ratio of methanol to oil and catalyst loading, which influence the biodiesel yield, which is regarded as the response factor. In RSM using a central composite design (CCD), the experimental data is evaluated and retrieved with a mathematical model for statistical analysis. This will help in the interpolation and extrapolation for the current system. The *p*-value test was considered to select the proper order of the mathematical model for the specific experimental data, and it should be less than 0.05, which shows its suitability with a 95% precision [35]. As shown in Table 4, it can be seen that the *p*-value for the two models was less than 0.05; thus, in this case, the highest order polynomial was selected. Therefore, for the current experimental data, the linear and quadratic model had a *p*-value less than 0.05. Therefore, the quadratic mathematical model was considered the most suitable for this case, which helped interpolate and extrapolate the data. Moreover, based on the predicted model, a statistical analysis was also done.

The statistical analysis (analysis of variance ANOVA) for the experimental data presented in Table 5 was carried out, in which there were four independent variables (temperature—A, time—B, catalyst loading—C and the methanol-to-oil molar ratio—D) against one response factor (biodiesel yield). As suggested earlier, for the current research work, a quadratic model fit well with the experimental data by the statistical analysis; the significance of the model was determined, along with their products (AB, AC, AD, BC and CD) and quadratic forms (A^2^, B^2^, C^2^ and D^2^), as shown in Table. The significance of the suggested model and other terms was determined based on the *p*-value test. In the *p*-value test, the *p*-value should be less than 0.05, which suggests its significance with a 95% confidence level. Thus, the *p*-value for the proposed quadratic model was 0.006, which was less than 0.05, revealing that it was significant with more than a 95% confidence level. Therefore, this showed that the model fit well with the experimental data based on this and could be interpolated and extrapolated easily.

Similarly, let us move along the *p*-values for each independent variable and their different combinations (AB, AC, AD, BC, CD, A2, B2, C2 and D2). Some are significant, and some are significant and insignificant. It has been reported that values greater than 0.1000 indicate the model terms are not significant. If there are many nominal model terms (not counting those required to support hierarchy), model reduction may improve the model. Moreover, the statistical analysis also helped determine the coefficient values based on which final quadratic model is formed, as shown in Equation (1).


(2) $$\begin{array}{l} Yield=78.14+10.47A+2.71B-1.9C+5.56D-0.34AB+0.61AC\\ \;-0.26AD-0.02BC-0.63BD+0.05CD-0.38A^{2}-0.24B^{2}\\ \;+0.52C^{2}-6.32D^{2} \end{array}$$

**Figure 5:**
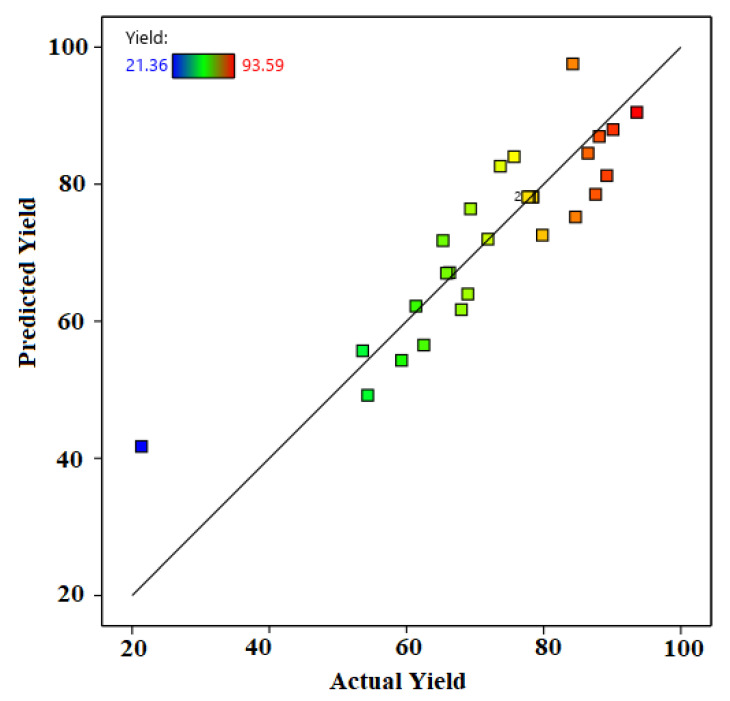
Correlation of the biodiesel yield obtained by the experiment (actual yield) and calculated results based on the model predicted by RSM (predicted yield).

The value of the response factor can be calculated based on the determined quadratic model. Thus, statistical analysis and model prediction can help extrapolate and interpolate the experimental data on the points other than the one on which the experiments were performed. So, this analysis, especially the model prediction, could help to commercialise the process. A plot was drawn between the actual yield (experimental) and predicted yield (model) to determine the fitting of the experimental data with the model-based data, as shown in Figure 5. Thus, based on the plot, the response factor had an experimentally calculated value (yield) that correlated with the yield calculated based on the predicted quadratic model. Therefore, based on the plot, it can be suggested that the predicted model was suitable and could help to scale up the process.

### 3.4. Parametric Study

**Figure 6:**
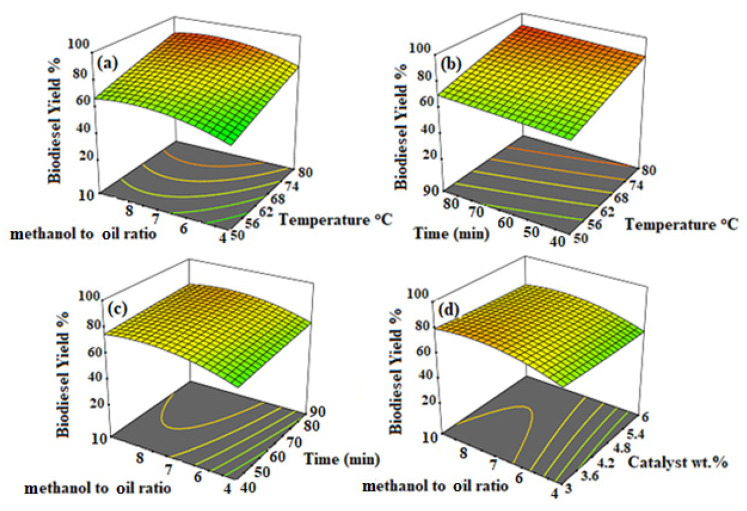
Process parameters are analysed for the biodiesel yield. (**a**) Effect of the process temperature and methanol-to-oil ratio on the biodiesel yield, (**b**) effect of the process temperature and time on the biodiesel yield, (**c**) effect of the process time and methanol-to-oil ratio on the biodiesel yield and (**d**) effect of the process catalyst loading and methanol-to-oil ratio on the biodiesel yield.

The biodiesel yield as a response factor against independent variables is shown in Figure 6. The plot shows the simultaneous combined effect of two independent variables against biodiesel yield, which helps to get more elaboration on the independence effectiveness. Figure 6a shows the effect of the process temperature and methanol-to-oil molar ratio on the biodiesel yield. It can be seen that, initially, at the lowest values of temperature (50 °C) and the molar ratio of oil to methanol (4), the biodiesel yield is relatively less; however, as we move along, the temperature and molar ratio of methanol to oil in the plot trends in a positive direction. The increment in biodiesel yield by increasing the temperature is because higher temperatures make the reactant molecules more excited, which possess enough energy at a higher temperature that they transform easily to biodiesel [36]. Similarly, the effect of the methanol-to-oil molar ratio is shown along with temperature on the biodiesel yield. It happens to increase when the molar ratio of methanol to oil increases.

Based on stoichiometric calculations, the molar ratio of methanol to oil should be three, and transesterification is a reversible reaction, thus avoiding a backwards reaction ratio. Initially, the biodiesel yield at the initial ratio of methanol to oil (4:1) is low, as shown in Table 2, due to less methanol availability in the reaction vessel. Moreover, increasing the amount of methanol tends to accelerate the transesterification reaction towards the methyl ester formation. However, excess methanol also increments monoglyceride formation, an intermediate product that causes the separation of the end product (FAME) from the by-product [37]. It can be observed from the trend that a maximum yield of 93.59% was achieved when the temperature reached 80 °C and a methanol-to-oil molar ratio of 8.

Further on, a slight decrement in the biodiesel yield was observed on the axial point (temperature), as shown in Table 2. A higher temperature beyond 80 °C may lead to less methanol availability in a reactor, causing fewer methyl ester formations (biodiesel). It was observed that a maximum biodiesel yield of 93.59% was obtained when the methanol-to-oil molar ratio was 8. Further on, when the methanol-to-oil molar ratio increased beyond 8, the biodiesel’s slight decrement is observed at an axial point, as shown in Table 2. The decrement in the biodiesel yield at a higher ratio can cause difficulty in the downstream process. It becomes challenging to have a distinctive difference between the product biodiesel and by-product glycerol.

Figure 6b shows the simultaneous effect of the process time and temperature on the biodiesel yield. The processing time was related to a residence time of reaction, which included all the factors, such as interaction duration reactants with the catalyst, reactants with each other and products with reactants. So, initially, when the reaction time and temperature were less, the yield was also less, depicting that enough of an interaction was not allowed between reactants and reactants with active catalyst sites. Therefore, when the time and temperature were increased, the yield rose until it reached a maximum of 90 min and 80 °C, which was thought to be a critical point at which the interaction between the reactant and the active catalyst sites became the optimal maximum biodiesel yield of 93.59%. After the critical points, the biodiesel started decreasing, as shown in Table 2 at the axial point (Time), which could be related to facts such that, at a higher residence time, the interaction between the reactants and products increased, which may lead to phase emergence and cause difficulty in product separation [38].

Figure 6c shows the simultaneous effect of the process time and methanol on the biodiesel yield’s oil molar ratio. It can be observed that both factors influenced the biodiesel yield initially at the start; with a lesser methanol-to-oil ratio (3) and time (40 min), the biodiesel yield was less. However, by increasing both parameters, the biodiesel yield also showed increment, just as it made it possible for the reactants to react with enough methanol availability. Further on, Figure 6d shows the effect of catalyst loading and the methanol-to-oil ratio simultaneously on the biodiesel yield when the other two parameters are kept constant. Catalyst loading significantly affected the biodiesel yield, as it referred to the amount of active site availability in the reaction vessels.

Moreover, as the catalyst is heterogeneous up to a specific limit, it can be beneficial for reactants and reaction mixtures, as a higher amount of catalyst loading may cause adverse effects to the reaction [39]. The issues related to higher catalyst loading are that it can cause a mass transfer limitation and cause a problem in the agitation of the reaction mixture, which causes adverse effects on the reaction. Therefore, it can be observed that the maximum biodiesel diesel is obtained when the catalyst loading is 3 wt.%, and when increasing beyond this amount, the biodiesel yield started decreasing.

The biodiesel yield was studied against the four process parameters discussed and found that the selected parameters had a significant effect. The optimal process parameters were: process temperature 80 °C, process time 90 min, methanol-to-oil molar ratio 8 and catalyst loading 3 wt.%. The optimal biodiesel yield was 93.59% on the optimal set of parameters. It can be observed that, when a heterogeneous catalyst was used for biodiesel production, the reaction time was quite (120–240 min) high due to the phase difference to get the maximum biodiesel yield. Similarly, it was also relatively high (9–18) for the methanol-to-oil molar ratio to complete triglycerides into methyl esters (biodiesel). Therefore, in the current study, as shown in the parametric analysis, the reaction time and methanol-to-oil molar ratio were relatively less, which could be related to the fact that the synthesised catalyst acted as highly active due to the high alkalinity, as shown in Table 3, due to addition of Cs_2_O as the active alkali oxide. Thus, the synthesised catalyst tends to give a better biodiesel yield without extreme processing conditions.

### 3.5. Reusability

**Figure 7:**
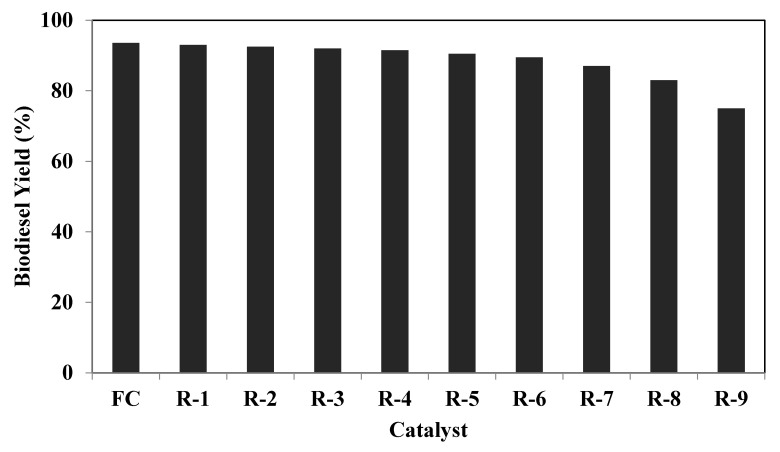
The catalyst reusability for the biodiesel yield for a fresh catalyst (FC) and used up to 10 experimental runs.

Industrial or commercial scale feasibility comes when a material is synthesised to be used as a catalyst for some chemical reaction, followed by its suitability in terms of a high product yield. The consumption or demand of the commercial scale of a catalyst depends on its reuse, which ultimately ends with an economical process. Therefore, to examine the feasibility and suitability of the synthesised catalyst, to use it on a commercial scale, reusability studies were carried out where the EC-15 catalyst was used up to ten repeated reactions, and the results in the biodiesel yield are shown in Figure 7. After each experimental run, the used catalyst was recovered and thoroughly washed with methanol to remove any reaction mixture and then dried in an oven at 120 °C for 4 h and reused for the following reaction cycle to produce biodiesel. Based on this pre-treatment of the used catalyst before each reaction, no intensive treatment was required. It was as active as used in the previous biodiesel reaction, in terms of biodiesel, up to eight consecutive transesterification reactions, as shown in Figure. In the ninth reaction cycle, the biodiesel yield was reduced to some extent, and in the tenth reaction, there was a noticeable decrement in the biodiesel yield. It was reported earlier that, when a heterogeneous catalyst was used for a particular chemical reaction upon its reuse, its activity became less towards the reaction due to the active site’s deactivation [32].

The reusability of the catalyst was also monitored by the conducted EDS analysis for the used catalyst after each reaction cycle, and the results are mentioned in Table 6. The EDS results helped monitor the active elements that offered active sites for the transesterification reaction of PPSS oil to produce biodiesel. It can be seen that, initially, up to the fourth reaction, the elements’ composition is not altered as much compared to the fresh catalyst. However, the yield started decreasing but was significantly less, as shown in Figure 7 in the seventh reaction. Still, a sudden evident decrement in the biodiesel yield was observed in the ninth reaction and more in the tenth reaction. Thus, when these results are compared to the EDS results of a catalyst recovered from the seventh reaction, the elemental composition observed a more pronounced change when the results for the eighth and onward reaction catalysts were monitored. Thus, these results predicted that the synthesised EC-15 catalyst was highly active for biodiesel production up to eight consecutive transesterifications, which showed that the synthesised catalyst is a promising catalyst for biodiesel production and can be scaled up for commercial biodiesel production in an economical way.

### 3.6. Properties of Biodiesel

Biodiesel produced from PPSS oil was evaluated based on its measured fuel properties (density, viscosity, cetane number, flash point, calorific value, pour point, cloud point, cold filter plugging point and water content) and compared to standards such as the ASTM and EN. The measured fuel properties followed by their comparisons with the standard values given by the ASTM and EN revealed the feasibility and potential of a new biomass feedstock and novel synthesised catalyst EC-15 to report the potential fuel (biodiesel) production, which can be scaled up for commercial production. First of all, the biodiesel density was measured at 25 °C and 884 kg/m^3^, satisfying the standard value (EN14214 gave the range 860–900 kg/m^3^). Density affects the fuel efficiency in terms of the quantity of fuel injected and effective combustion [40]. Therefore, based on this observation, it can be reported that the amount of biodiesel is essential to be measured, and for efficient fuel, it should satisfy the standard limits. Then, the viscosity of the produced biodiesel was measured at 40 °C, and its value was found to be 3.91 mm^2^/s, satisfying the standard’s defined limits (ASTM6751 limit is 1.9–6.0 mm^2^/sec, and EN14214 limit is 3.5–5.0 mm^2^/s). The viscosity of the fuel used for combustion directly affects its injection into the combustion chamber in the engine; thus, it should be controlled and monitored properly to have efficient fuel [41].

The fuel needs to be stored and transported once produced; thus, it must be exposed to a diversified atmosphere. Therefore, to have efficiency in terms of its transportation and storage so as it does not explode, one of the significant properties is measured in this regard: the flashpoint of fuel [38]. It is the maximum temperature that fuel can bear without catching flame. In the biodiesel produced in the current research work, the PPSS oil flashpoint was 167 °C, and it satisfied the limits given by the ASTM6751 (93 °C minimum) and EN14214 (120 °C minimum) standards. Therefore, the produced biodiesel is safe to be stored and transport, as it satisfies the standards. The cetane number of the biodiesel was 62.14, while the minimum value as per the standards was ASTM6751-47 and EN14214-51. Thus, the cetane value measurement and its comparison with the standards revealed that biodiesel has a good combustion reaction capability, enhancing the fuel efficiency and engine life [42]. The energy density of the produced biodiesel was measured in terms of its calorific value, 44.12 MJ/kg. The measured calorific of the produced biodiesel was almost similar to the fossil diesel fraction, thus predicting that the produced biodiesel can be efficiently used in conventional diesel engines without any prior modifications.

The fuel is considered efficient when used in diversified environmental conditions, and one of the negative points regarding biodiesel is its poor low-temperature properties [10]. Low-temperature properties are dependent on the composition of biodiesel. If a biodiesel is composed of more saturated methyl esters, it will give poor low-temperature properties. Thus, this kind of biodiesel becomes inefficient for cold regions and weather, as its saturated components tend to solidify at high temperatures compared to the unsaturated components. The biodiesel from PPSS oil contains more unsaturated methyl esters compared to the saturated methyl esters, as mentioned in Table 1, which results in better lower temperature properties such as the cloud point (1.3 °C), pour point (−4.7 °C) and cold filter plugging point (−3.1 °C). Thus, based on low-temperature properties, it can be recommended that biodiesel produced from PPSS oil can be suitable as fuel in cold regions and weather without difficulty. The last water content of producing biodiesel was measured, and it had a ≤0.01 volume percent, and the standard value as per ASTM6751 is 0.05 volume percent. This revealed that the water content in biodiesel produced from PPSS oil is almost negligible and can efficiently be used in conventional diesel. The higher water content in fuel could cause a decrement in engine efficiency. Therefore, based on biodiesel’s fuel properties obtained from PPSS oil using a novel heterogeneous catalyst, it can be confidently reported that biodiesel is highly efficient; PPSS oil can be a flow potential alternative to the EC-15 highly efficient heterogeneous catalyst for transesterification reactions.

## 4. Conclusions

The present study revealed that waste eggshells could be used as a potential source for synthesising a heterogeneous catalyst, which was further enhanced by adding caesium oxide as the active promoter material. Due to its abundant availability and oil presence, *Pongamia pinnata* seed shells (PPSS) tend to be a potential biodiesel production source. Further on, PPSS oil’s transesterification in the presence of a synthesised catalyst that was novel to its kind gave a high biodiesel yield of 93.59%. Thus, based on the parametric study, it has been found that the processing time and methanol-to-oil ratio while using a highly basic synthesised catalyst are reduced as much as 90 min and eight, respectively, which are almost similar to the conditions when a homogeneous catalyst is used for biodiesel production. The synthesised catalyst is also reusable, which shows its potential to be commercialised. Moreover, the fuel properties of biodiesel were according to the limits defined by the ASTM6751 and EN14214 standards.

## Figures and Tables

**Table 1:** Process parameters for biodiesel and its experimental ranges.

Range	Temperature (°C)	Methanol-to-Oil Molar Ratio	Time (min)	Catalyst Loading (wt.%)
Axial point	35	2	15	1.5
Low	50	4	40	3
Mid	65	6	65	4.5
High	80	8	90	6
Axial point	95	10	115	7.5

**Table 2:** Experimental plan, along with biodiesel yield.

Exp. Run	Temperature (°C)	Time (Minutes)	Catalyst (wt.%)	Methanol:Oil Ratio	Biodiesel Yield (%)
1	80	90	6	8	90.12
2	50	40	6	4	54.35
3	65	65	4.5	6	77.89
4	50	90	6	4	62.52
5	50	90	3	8	71.87
6	80	40	6	8	86.47
7	80	90	3	8	93.59
8	35	65	4.5	6	53.61
9	65	65	4.5	6	78.12
10	80	90	6	4	87.58
11	80	40	3	4	84.65
12	80	90	3	4	89.21
13	65	65	4.5	6	78.37
14	80	40	6	4	79.82
15	65	65	7.5	6	69.34
16	65	65	1.5	6	75.69
17	50	40	3	4	59.29
18	65	65	4.5	6	78.39
19	80	40	3	8	88.11
20	50	90	3	4	68.01
21	65	15	4.5	6	65.32
22	65	115	4.5	6	73.68
23	65	65	4.5	6	77.69
24	50	40	3	8	66.29
25	65	65	4.5	2	21.36
26	65	65	4.5	6	78.39
27	50	90	6	8	65.84
28	95	65	4.5	6	84.25
29	65	65	4.5	10	68.95
30	50	40	6	8	61.38

**Table 3:** Basicity analysis.

Catalyst	Basicity (mmol/g)
EC	3.15 ± 0.14
EC-10	5.96 ± 0.26
EC-15	6.46 ± 0.11
EC-20	4.86 ± 0.12
EC-25	5.17 ± 0.10

EC: lime (CaO) derived from waste eggshells (EC), EC-10: lime modified by 10 wt.% of caesium oxide, EC-15: lime (CaO) modified by 15 wt.% of caesium oxide, EC-20: lime (CaO) modified by 20 wt.% of caesium oxide and EC-25: lime (CaO) modified by 25 wt.% of caesium oxide.

**Table 4:** Model prediction for analysing based on the experimental data for biodiesel production.

Source	Sum of Squares	df	Mean Square	F-Value	*p*-Value
Mean vs. Total	1.599 × 10^5^	1	1.599 × 10^5^		
Linear vs. Mean	3635.98	4	909.00	9.18	0.0001
2FI vs. Linear	15.32	6	2.55	0.02	1.000
Quadratic vs. 2FI	1162.27	4	290.57	3.36	0.037
Cubic vs. Quadratic	586.27	8	73.28	0.72	0.674
Residual	712.40	7	101.77		
Total	1.660 × 10^5^	30	5533.47		

**Table 5:** ANOVA for the predicted model and for each experimental process parameter.

Source	Sum of Squares	df	Mean Square	F-Value	*p*-Value
Model	4813.58	14	343.83	3.97	0.006
A—Temperature	2630.90	1	2630.90	30.39	<0.0001
B—Time	176.58	1	176.58	2.04	0.173
C—Catalyst	86.79	1	86.79	1.00	0.332
D—Me:Oil	741.70	1	741.70	8.57	0.010
AB	1.88	1	1.88	0.021	0.884
AC	6.00	1	6.00	0.069	0.795
AD	1.09	1	1.09	0.012	0.912
BC	0.005	1	0.005	0.0001	0.993
BD	6.30	1	6.30	0.072	0.791
CD	0.044	1	0.044	0.0005	0.982
A^2^	3.94	1	3.94	0.045	0.834
B^2^	1.53	1	1.53	0.017	0.895
C^2^	7.34	1	7.34	0.084	0.774
D^2^	1096.50	1	1096.50	12.66	0.002

**Table 6:** EDS analysis of the fresh catalyst (FC) and its used form after each experimental run (R-1–R-9).

Run	Catalyst	Calcium (%)	Caesium (%)
1	FC	71.89	14.89
2	R-1	71.88	14.84
3	R-2	71.81	14.77
4	R-3	71.78	14.71
5	R-4	71.60	14.64
6	R-5	71.55	14.51
7	R-6	71.40	14.06
8	R-7	70.96	13.38
9	R-8	70.10	12.31
10	R-9	69.03	11.19

## Data Availability

Not applicable.

## Sample Availability

Samples of the compounds Cs_2_O are available from the authors.
